# Abnormal Calcium Metabolism Mediated Increased Risk of Cardiovascular Events Estimated by High Ankle-Brachial Index in Patients on Peritoneal Dialysis

**DOI:** 10.3389/fcvm.2022.920431

**Published:** 2022-07-28

**Authors:** Xiaoyan Su, Wanbing He, Mengbi Zhang, Yinyin Zhang, Langjing Zhu, Jie Chen, Hui Huang

**Affiliations:** ^1^Department of Nephrology, Dongguan Tungwah Hospital, Dongguan, China; ^2^Department of Cardiology, Sun Yat-sen Memorial Hospital, Sun Yat-sen University, Guangzhou, China; ^3^Department of Nephrology, The Eighth Affiliated Hospital, Sun Yat-sen University, Shenzhen, China; ^4^Department of Radiation Oncology, Sun Yat-sen Memorial Hospital, Sun Yat-sen University, Guangzhou, China; ^5^Department of Cardiology, The Eighth Affiliated Hospital, Sun Yat-sen University, Shenzhen, China

**Keywords:** ankle-brachial index, peritoneal dialysis, calcium, vascular calcification, Kt/V

## Abstract

Cardiovascular disease (CVD) is the leading cause of death in peritoneal dialysis (PD) patients. But the relationship between regular PD and the risk of major adverse cardiovascular events (MACE) remains controversial. The possible risk factors are not fully elucidated. This study aims to investigate the possible factors affecting the risk of MACE estimated by high ankle-brachial index (ABI) in PD patients. A total of 243 patients were enrolled and divided into chronic kidney diseases (CKD) stage 1, non-dialyzed CKD stages 2–5, and PD groups. The prevalence of high ABI, indicating increased MACE, was elevated with CKD progression but not further increased in PD patients. Systolic blood pressure was closely correlated with high ABI in non-dialyzed CKD patients (β = 0.059, *P* = 0.001). But in PD patients, serum calcium had a crucial effect on high ABI (β = −9.853, *P* < 0.001). Additionally, PD patients with high ABI tended to dialyze inadequately (Kt/V <1.7) compared to those with normal ABI (29.0 vs. 13.3%, *P* = 0.031). Further mediation analysis revealed that ~86.2% of the relationship between Kt/V and high ABI was mediated by serum calcium in PD patients (mediation effect = 86.2%, ab = −0.220, 95% CI: −0.381 to −0.059, *P* = 0.008), especially in those starting PD before 55 years of age and with normal body mass index. This present study indicated that improvement of PD adequacy by maintaining calcium balance might be a promising method to reduce the risk of MACE estimated by high ABI for PD patients.

## Introduction

Chronic kidney disease (CKD) is a substantial burden threatening global health (1). Among the causes of death, cardiovascular disease (CVD) is still the leading one of CKD death, which is increased with the development of CKD stages and is even up to 10–20 times higher in dialysis patients than in the general population (2, 3). Therefore, CVD prevention is important for CKD, especially for patients on dialysis. Since traditional risk factors such as hypertension and hypercholesterolemia cannot fully explain the high mortality of CVD in the CKD population, untraditional risk factors, especially vascular calcification (VC), have caused great concern recently (4). Defined as the deposition of extraosseous calcium (Ca) in the vasculature, VC is a common complication in CKD patients (5). Studies have demonstrated that VC is strongly associated with CVD mortality in patients with CKD (6–8). Ankle-brachial index (ABI) is a simple and non-invasive method widely used in clinical practice to identify peripheral artery disease. High ABI (>1.3) indicates artery stiffness and has a close relationship with VC. It is reported that high ABI is independently associated with major adverse cardiac events (MACE) (9, 10), but remain unclear in dialysis patients. Adragao et al. reported that CKD patients with high ABI exhibited nearly 7-fold higher risk of cardiovascular mortality (11). Therefore, ABI is a promising indicator for VC and provides a better portable CVD risk assessment in CKD patients.

The state of uremic milieu is shown to be involved in the development of VC (5). However, patients with different CKD stages have different risk factors for VC development (12). Especially for dialysis patients, besides the common risk factors similar to non-dialyzed patients, other factors like hemodynamic stability and mineral metabolism also play important roles in VC as well (11, 13). Peritoneal dialysis (PD) is considered a relatively better choice for patients with end-stage renal disease than hemodialysis (HD) as its preference for hemodynamic stability and volume regulation (13). Regular PD is demonstrated to reduce mortality, but its effect on cardiovascular risks remains controversial (14). Moreover, the possible risk factors are not fully elucidated.

Therefore, we conducted a cross-sectional study to explore the risk factors affecting the risk of MACE evaluated by ABI among patients with different CKD stages or PD treatment. Then, we further investigated the possible mediators for high ABI in PD patients and sought possible strategies.

## Methods

### Study Population

This was a single-center observational study of consecutive CKD patients who were referred to the Tungwah Hospital of Sun Yat-sen University between November 2015 and November 2016. A total of 243 patients were enrolled in this study. All patients must undergo the ABI test.

The e-GFR of all included patients were calculated based on CKD-EPI equation (15). According to the CKD diagnostic criteria, patients were divided into three groups based on their e-GFR levels (1). Patients with the estimated-glomerular filtration rate (e-GFR) <15 mL/min per 1.73 m^2^ and receiving the regular continuous ambulatory peritoneal dialysis (CAPD) treatment for more than 3 months were defined as the PD group. Initiation of dialysis usually depended on the patient's preferences and medical necessity. It will be considered when one or more of the following are present: symptoms or signs attributable to kidney failure (e.g., neurological signs and symptoms attributable to uremia, pericarditis, anorexia, medically resistant acid-base or electrolyte abnormalities, reduced energy level, weight loss with no other potential explanation, intractable pruritus, or bleeding); inability to control volume status or blood pressure; and a progressive deterioration in nutritional status refractory to interventions (16, 17). Non-dialyzed patients with e-GFR <90 mL/min per 1.73 m^2^ were defined as non-dialyzed CKD stages 2–5 group, and the other patients who had the evidence of renal structural or functional abnormalities but e-GFR ≥ 90 mL/min per 1.73 m^2^ were defined as CKD stage 1 group.

Clinical data were extracted from the hospital database. Individuals with age <20 years, familial hyperlipidemia, severe hepatic dysfunction, carcinoma, potential infectious or inflammatory diseases, autoimmune diseases, peripheral artery disease, corticosteroid therapy, either one leg with ABI ≤ 0.9 were excluded from this study. In the PD group, patients who had peritonitis in the past 3 months and cannot receive CAPD should be excluded.

The study protocol conformed to the ethical guidelines of the 1975 Declaration of Helsinki as reflected in *a priori* approval by the Ethics Committee of the Tungwah Hospital of Sun Yat-sen University. Informed consent was obtained from each participant.

### Laboratory Parameters

Laboratory parameters were all measured by using blood samples. Each patient should fast overnight for at least 10 h before venipuncture. Biochemical parameters, Ca, serum phosphorus (P), creatinine, alkaline phosphatase (ALP), fasting plasma glucose (FPG), glycated hemoglobin (HbA1c), total cholesterol (TC), high-density lipoprotein cholesterol (HDL-C), low-density lipoprotein cholesterol (LDL-C), triglyceride (TG), cystatin C (CysC), etc. were analyzed by a standardized and certified TBA-120 auto-analyzer (Toshiba Medical Systems, Japan) in the institutional central laboratory.

### Dialysis Adequacy

To estimate dialysis adequacy, weekly Kt/V was calculated based on 24-h urine performed prior to the scheduled visit to the PD unit. Weekly Kt/V values were calculated according to the standard method recommended in the kidney disease outcomes quality initiative guidelines (18). The cut-off value of Kt/V was 1.7, as recommended by K/DOQI guidelines (19). Kt/V ≥ 1.7 indicated adequate PD while Kt/V <1.7 indicated insufficient PD.

### Measurement of ABI

Ankle-brachial index was measured by a non-invasive vascular screening device (VP-1000, OMRON, Japan) that was reported before (20, 21).

### Definition of Adverse Cardiovascular Events

History of adverse cardiovascular events was collected including cardiac death, coronary heart disease (CHD), congestive heart failure (CHF), acute myocardial infarction (AMI), and acute cerebral infarction (ACI). CHD was defined as ≥50% diameter stenosis of coronary arteries by either coronary angiography or CT angiography (22). CHF was diagnosed according to 2016 ESC Guidelines for the diagnosis and treatment of chronic heart failure (23). AMI was diagnosed according to 2015 ESC Guidelines for the management of acute coronary syndromes (24). ACI was defined as an acute neurological event lasting more than 24 h associated with the evidence of ischemic focus of the brain in computer tomography or magnetic resonance imaging. Cardiac death was defined as death caused by AMI, arrhythmias, or CHF. The combination of CHD, CHF, AMI, ACI, and cardiac death was defined as MACE in this study.

### Statistical Analysis

Data were presented as frequencies for categorical variables, mean values with standard deviation (SD) for normally distributed continuous variables, and median values with 25 and 75% percentiles for ordinal variables. The group differences among different CKD-stage groups were assessed by the analysis of variance (ANOVA) or Pearson chi-square according to the data types. Independent risk factors for high ABI were identified by binary logistic regression with the forward conditional method.

Mediation analysis was conducted to explore the mediator of the relationship between age, e-GFR or Kt/V, and high ABI. Mediation existed when four conditions were met: first, the predictor (in this case age, e-GFR or Kt/V) must have a significant relationship with the outcome variable (high ABI) in pathway c; second, the predictor must also have a significant relationship with the potential mediators in pathway a; third, the mediator must have a significant relationship with the outcome when the effect of the predictor on the outcome was controlled for pathway b; fourth, the relationship between predictor and outcome must be decreased (lower than in pathway c) when controlling for the mediators in pathway c′. If the predictor remained significant when the mediator was controlled for, the mediation was considered partial. When controlling for the mediator rendered the predictor non-significant, mediation was considered complete. In this article, both ABI (ABI ≥ 1.3 as 1 and 0.9 < ABI <1.3 as 0) and Kt/V (Kt/V ≥ 1.70 as 1 and <1.70 as 0) were dichotomous variances while age, e-GFR, and all mediators were continuous. Ordinary least squares regression or logistic regression might be chosen according to the types of variances. Therefore, the parameter estimates a, b, c, c′ and their standard errors calculated above must be standardized according to the methods suggested by MacKinnon. Then the indirect effect was calculated by standardized parameters and tested for significance by the Sober test. The mediated effect size was also evaluated by a formula ab/(ab + c′), where a, b, and c' were all standardized (25).

All statistical analyses were performed using the software SPSS version 22.0 (SPSS, Inc, Chicago, IL), and 2-sided *P*-values <0.05 were considered statistically significant.

## Results

### Comparison of Clinical Characteristics Among CKD Stage 1, Non-dialyzed CKD Stages 2–5, and PD Groups

The clinical characteristics of CKD stage 1, non-dialyzed CKD stages 2–5, and PD groups are shown in Table 1. The PD patients tended to be smokers and exhibited higher rate of hypertension with higher blood pressure than the other groups (smoking: 39.5 vs. 9.1 vs. 7.7%, *P* < 0.001; hypertension: 87.7 vs. 48.1 vs. 64.9%, *P* < 0.001; SBP: 152 ± 21 mmHg vs. 135 ± 20 mmHg vs. 126 ± 23 mmHg, *P* < 0.01; and DBP: 89 ± 13 mmHg vs. 82 ± 15 mmHg vs. 82 ± 15 mmHg, *P* < 0.001). Moreover, the levels of P and CysC were increased with the elevation of CKD stages and reached the highest in PD patients (P: 1.10 ± 0.19 mmol/L vs. 1.11 ± 0.23 mmol/L vs. 1.73 ± 0.54 mmol/L, *P* < 0.001; CysC: 0.91 ± 0.21 mg/L vs. 1.53 ± 0.83 mg/L vs. 5.80 ± 1.54 mg/L, *P* < 0.001), while Ca showed the opposite trend (2.30 ± 0.14 mmol/L vs. 2.24 ± 0.22 mmol/L vs. 2.16 ± 0.24 mmol/L, *P* < 0.001). However, no significant differences were found in age, sex ratios, body mass index (BMI), prevalence of diabetes mellitus (DM), the levels of FPG, TC, HDL-C, LDL-C, and TG (*P* > 0.05) (Table 1).

**Table 1:** Clinical characteristics of non-dialyzed CKD stages 1–5 and PD patients.

	**CKD stage 1**	**CKD stage 2–5 (Non-dialyzed)**	**PD**	***P*-value**
N	52	77	114	
Age (y)	43 ± 7	47 ± 13	44 ± 14	0.122
Male (%)	51.9	50.6	64.0	0.128
BMI (kg/m^2^)	22.7 ± 3.0	23.0 ± 5.0	22.1 ± 3.7	0.329
SBP (mmHg)	126 ± 23	135 ± 20	152 ± 21	<0.001*
DBP (mmHg)	82 ± 15	82 ± 15	89 ± 13	0.001*
Smoking (%)	7.7	9.1	39.5	<0.001*
Hypertension (%)	48.1	64.9	87.7	<0.001*
DM (%)	11.5	16.9	14.0	0.690
FPG (mmol/L)	5.20 ± 0.90	5.36 ± 1.16	5.22 ± 1.76	0.762
ALP (U/L)	76 ± 25	78 ± 25	73 ± 16	0.309
Ca (mmol/L)	2.30 ± 0.14	2.24 ± 0.22	2.16 ± 0.24	<0.001*
P (mmol/L)	1.10 ± 0.19	1.11 ± 0.23	1.73 ± 0.54	<0.001*
K (mmol/L)	4.08 ± 0.37	4.24 ± 0.52	4.04 ± 0.75	0.087
TC (mmol/L)	4.60 ± 0.81	4.88 ± 1.02	4.77 ± 1.25	0.366
LDL-C (mmol/L)	2.63 ± 0.78	2.93 ± 0.94	2.72 ± 1.01	0.170
HDL-C (mmol/L)	1.25 ± 0.38	1.28 ± 0.42	1.25 ± 0.39	0.874
TG (mmol/L)	1.73 ± 1.38	1.70 ± 0.90	1.72 ± 1.34	0.994
Cr (mmol/L)	70.2 ± 18.1	143.4 ± 113.1	979.7 ± 322.3	<0.001*
CysC (mg/L)	0.91 ± 0.21	1.53 ± 0.83	5.80 ± 1.54	<0.001*
e-GFR [ml/(min·1.73m^2^)]	111.7 ± 20.0	58.1 ± 22.7	–	<0.001*
Kt/V	–	–	2.04 ± 0.36	–
PD period (m)	–	–	38.8 ± 27.5	–
Residual renal function (KRU > min)			7.26 ± 3.18	
High ABI (%)	9.8	23.1	27.2	0.045*

ABI, ankle-brachial index; ALP, alkaline phosphatase; BMI, body mass index; Ca, calcium; CKD, chronic kidney disease; Cr, creatinine; CysC, cystatin C; DBP, diastolic blood pressure; DM, diabetes mellitus; e-GFR, estimated-glomerular filtration rate; FPG, fasting plasma glucose; HDL-C, high-density lipoprotein cholesterol; K, serum potassium; LDL-C, low-density lipoprotein cholesterol; P, phosphorus; PD, peritoneal dialysis; SBP, systolic blood pressure; TC, total cholesterol; TG, triglycerides; *P <0.05.

### The Prevalence of High ABI Was Increased With CKD Progression

**Figure 1:**
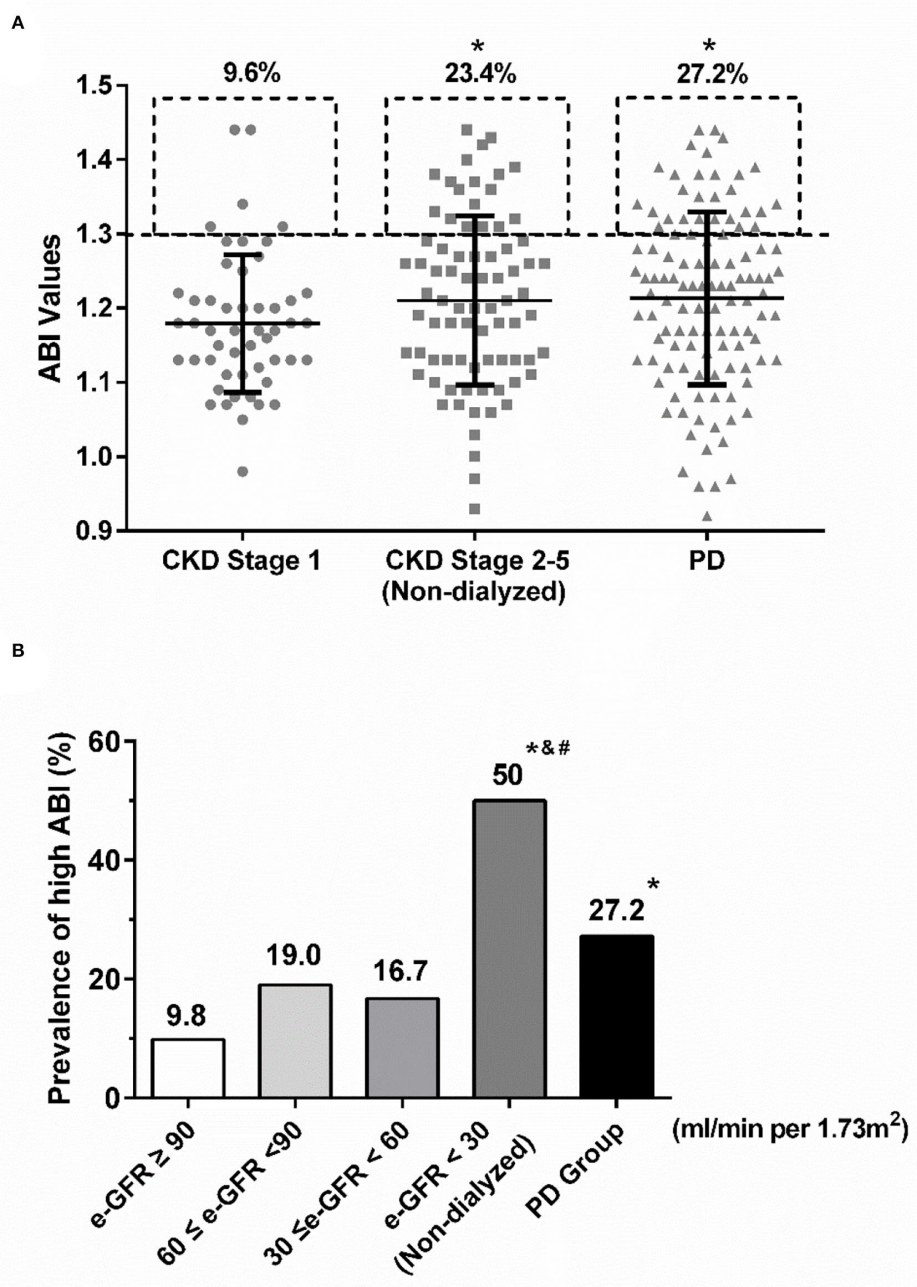
The prevalence of high ABI in various CKD stages. **(A)** The rates of high ABI were similarly increased in non-dialyzed CDK stage 2–5 and PD groups compared with CKD stage 1. *P < 0.05 vs. CKD stage 1 group. **(B)** Similar upgraded trend of high ABI was shown in the decline of e-GFR. However, the prevalence of high ABI in PD patients was much lower than that in non-dialyzed CKD patients with e-GFR <30 ml/min per 1.73m^2^. *P < 0.05 vs. the group with e-GFR ≥ 90 ml/min per 1.73 m^2^; # *P* < 0.05 *vs*. the group with 60 ≤ e-GFR <90 ml/min per 1.73 m^2^; and & *P* < 0.05 vs. the group with 30 ≤ e-GFR <60 ml/min per 1.73 m^2^. ABI, ankle-brachial index; CKD, chronic kidney disease; e-GFR, estimated-glomerular filtration rate; PD, peritoneal dialysis.

Prevalence of high ABI was significantly different among CKD stage 1, non-dialyzed CKD stages 2–5, and PD groups (9.6 vs. 23.4 vs. 27.2%, *P* = 0.045) (Figure 1A). Non-dialyzed CKD stages 2–5 and PD patients shared similar prevalence of high ABI and both were higher than that of CKD stage 1 patients (non-dialyzed CKD stage 2–5 vs. CKD stage 1: *P* = 0.054; PD vs. CKD stage 1: *P* = 0.012; non-dialyzed CKD stage 2–5 vs. PD: *P* = 0.521; Figure 1A). Furthermore, about 50% of non-dialyzed patients with e-GFR <30 ml/min per 1.73 m^2^ had high ABI (Figure 1B). Interestingly, the prevalence tended to decrease in PD patients compared with those with similar e-GFR but without dialysis (e-GFR <30 ml/min per 1.73 m^2^: 50.0% vs. PD: 27.2%, *P* = 0.477) (Figure 1B). However, no significant difference was observed in ABI values among various groups or e-GFR levels. The results showed that high ABI occurred more frequently with the decrease of e-GFR but the prevalence was not further increased in PD patients.

### Different Risk Factors Contributed to ABI Between PD and Non-dialyzed Patients

**Figure 2:**
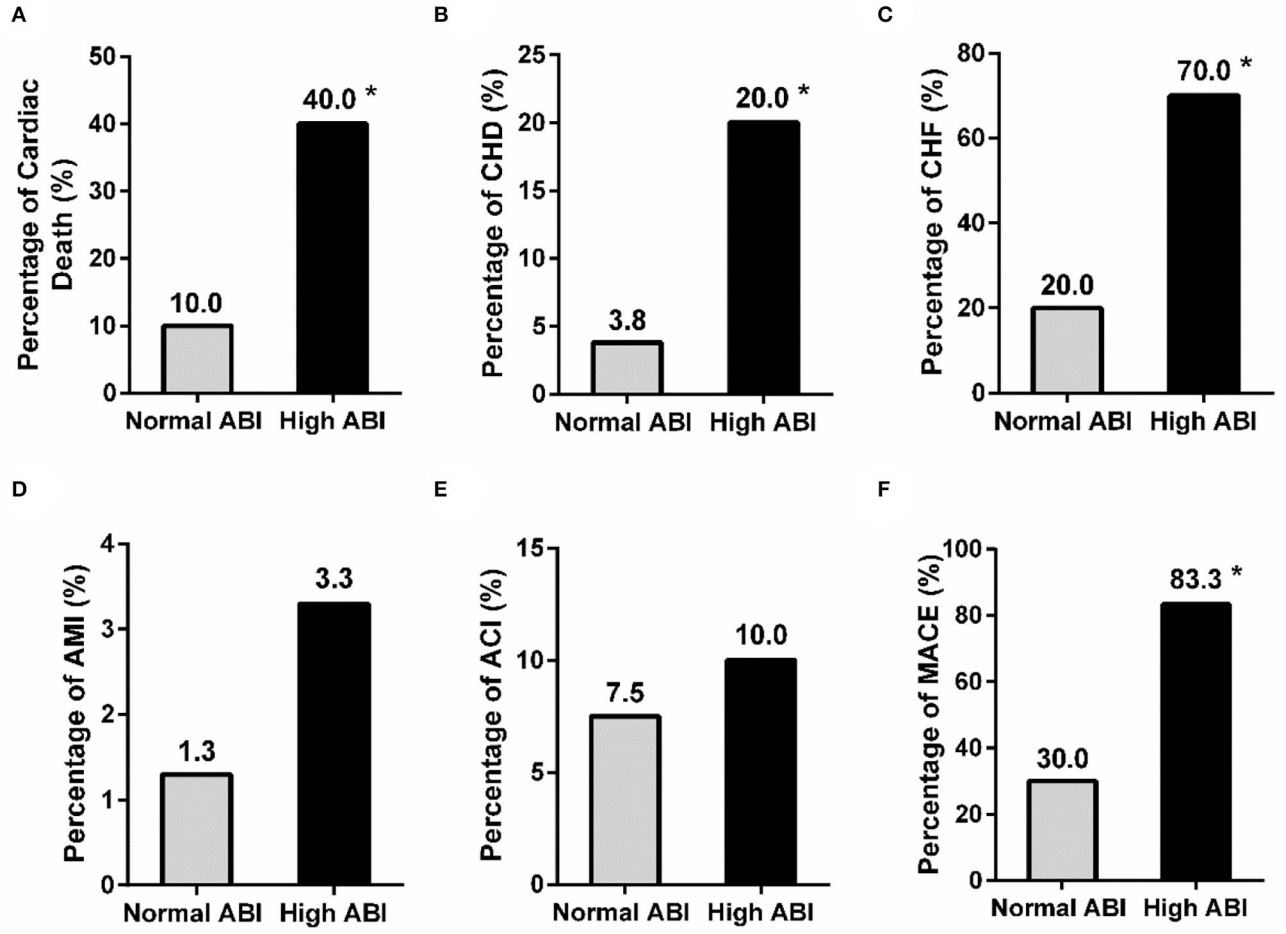
Comparison of the history of adverse cardiovascular events between the normal and high ABI groups in PD patients. The prevalence of cardiac death, CHD, and CHF **(A–C)**, but not AMI and ACT **(D,E)**, was significantly different between patients with normal and high ABI. A significant difference was also observed in the rate of MACE **(F)** between the two groups. *P < 0.05 vs. normal ABI group. ABI, ankle-brachial index; ACI, acute cerebral infarction; AMI, acute myocardial infarction; CHD, coronary heart disease; CHF, congestive heart failure; MACE, major adverse cardiac events.

As known, high ABI has been reported to be associated with a high risk of adverse CVD in non-dialyzed patients (10). Our results showed that PD patients with high ABI also suffered more cardiac death, CHD, CHF, and total MACE than those with normal ABI (cardiac death: 40.0 vs. 10.0%, *P* < 0.001; CHD: 20.0 vs. 3.8%, *P* = 0.012; CHF: 70.0 vs. 20.0%, *P* < 0.001; MACE: 83.3 vs. 30.0%, *P* < 0.001) (Figure 2). The prevalence of AMI and ACI tended to be increased in the high ABI group than those in the normal ABI group, although the difference was not significant (AMI: 3.3% vs. 1.3%, *P* = 0.473; ACI: 10.0 vs. 7.5%, *P* = 0.702) (Figure 2). This indicated that high ABI was closely associated with a high prevalence of CVD in PD.

To explore the possible risk factors affecting high ABI between PD and non-dialyzed CKD patients, binary logistic regression analysis was used. The results showed that TC was a risk factor for high ABI in CKD stage 1 group while SBP and DM were in the non-dialyzed CKD stages 2–5 group (TC: β = 4.305, SE = 1.747, *P* = 0.014; SBP: β = 0.058, SE = 0.018, *P* = 0.010; DM: β = 1.542, SE = 0.689, *P* = 0.025) (Table 2). In the PD group, both age and Ca were the risk factors for high ABI (age: β = 0.074, SE = 0.023, *P* = 0.001; Ca: β = −9.853, SE = 2.020, *P* < 0.001) (Table 2). Taken together, the results suggested that traditional cardiovascular risk factors contributed to high ABI in non-dialyzed CKD patients while the serum level of Ca played an important role in PD ones.

**Table 2:** The binary logistic regression analysis of the independent risk factors for high ABI.

**Groups**		**β**	**SE**	***P*-value**
CKD stage 1	TC	4.305	1.747	0.014*
CKD stage 2–5 (non-dialyzed)	SBP	0.058	0.018	0.010*
	DM	1.542	0.689	0.025*
PD	Age	0.074	0.023	0.001*
	Ca	−9.853	2.020	<0.001*

Binary logistic regression analysis with forward conditional method was used to assess the independent risk factors of high ABI in the CKD stage 1, non-dialyzed CKD stage 2–5 and PD groups. The factors used for analysis were age, sex, BMI, SBP, hypertension, DM, smoking, TC, LDL-C, Ca, Pi P, e-GFR. ^*^P <0.05. ABI, ankle-brachial index; BMI, body mass index; Ca, calcium; CKD, chronic kidney disease; DM, diabetes mellitus; e-GFR, estimated-glomerular filtration rate; PD, peritoneal dialysis; Pi P, phosphorus; SBP, systolic blood pressure; SE, standard error; TC, total cholesterol.

### The Mediation Effects of Serum Calcium on High ABI in CKD Patients

**Figure 3:**
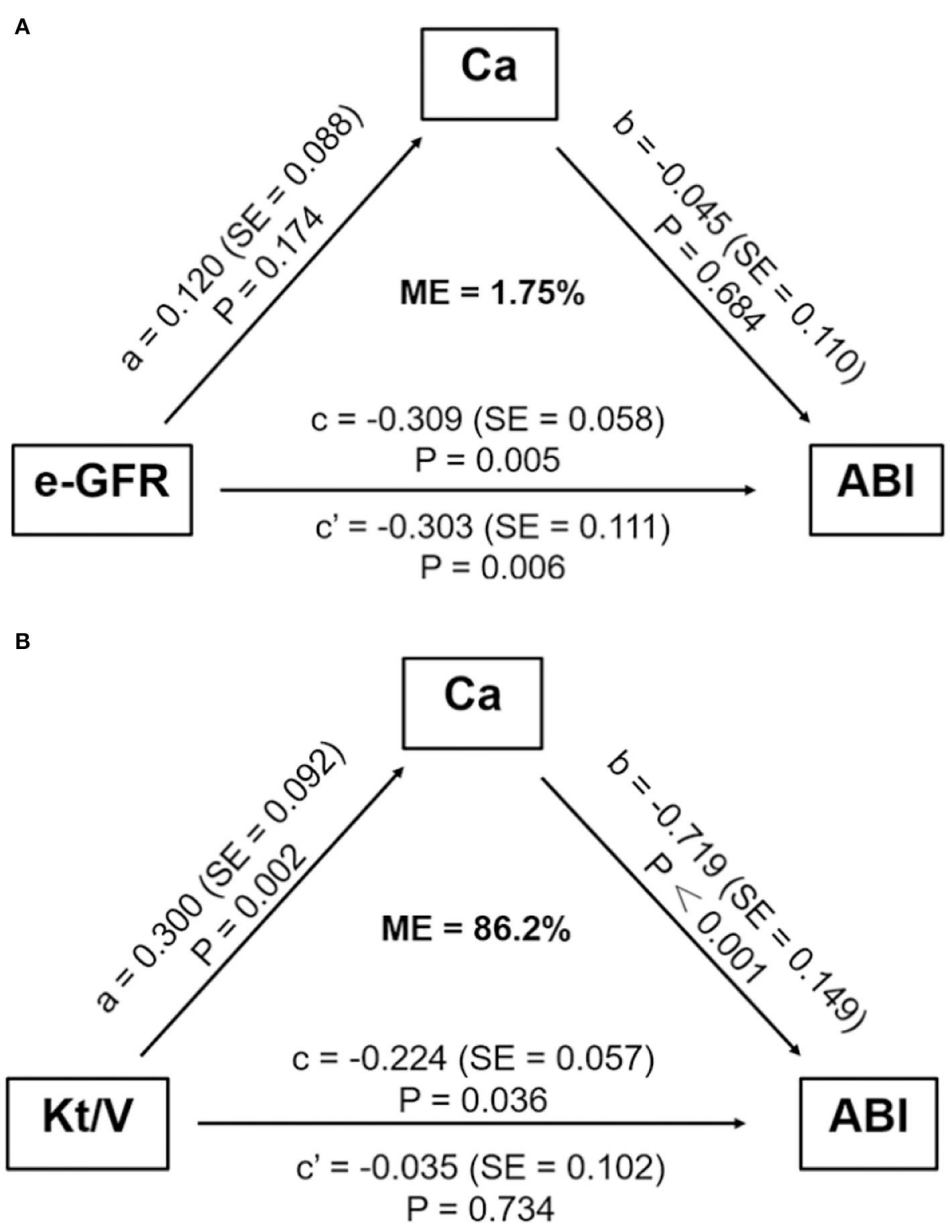
Mediation analysis on the association between e-GFR or Kt/V and ABI. **(A)** The mediation effect of serum calcium did not exist in the association between e-GFR and high ABI in non-dialyzed CKD patients. **(B)** Serum calcium mediated the association between Kt/V and high ABI in PD patients. ABI, ankle-brachial index; Ca, calcium; e-GFR, estimated-glomerular filtration rate; ME, mediation effect; SE, standard error.

We further studied whether any mediators functioned in the association between e-GFR and high ABI in non-dialyzed CKD patients. As shown in Figure 3A, indirect effect of Ca was not significant in the relationship between e-GFR and ABI for non-dialyzed CKD patients (ab = −0.005, 95% CI: −0.390 to 0.022, *P* = 0.696). This result indicated that Ca did not act as a mediator in the relationship between e-GFR and high ABI in non-dialyzed CKD patients.

**Figure 4:**
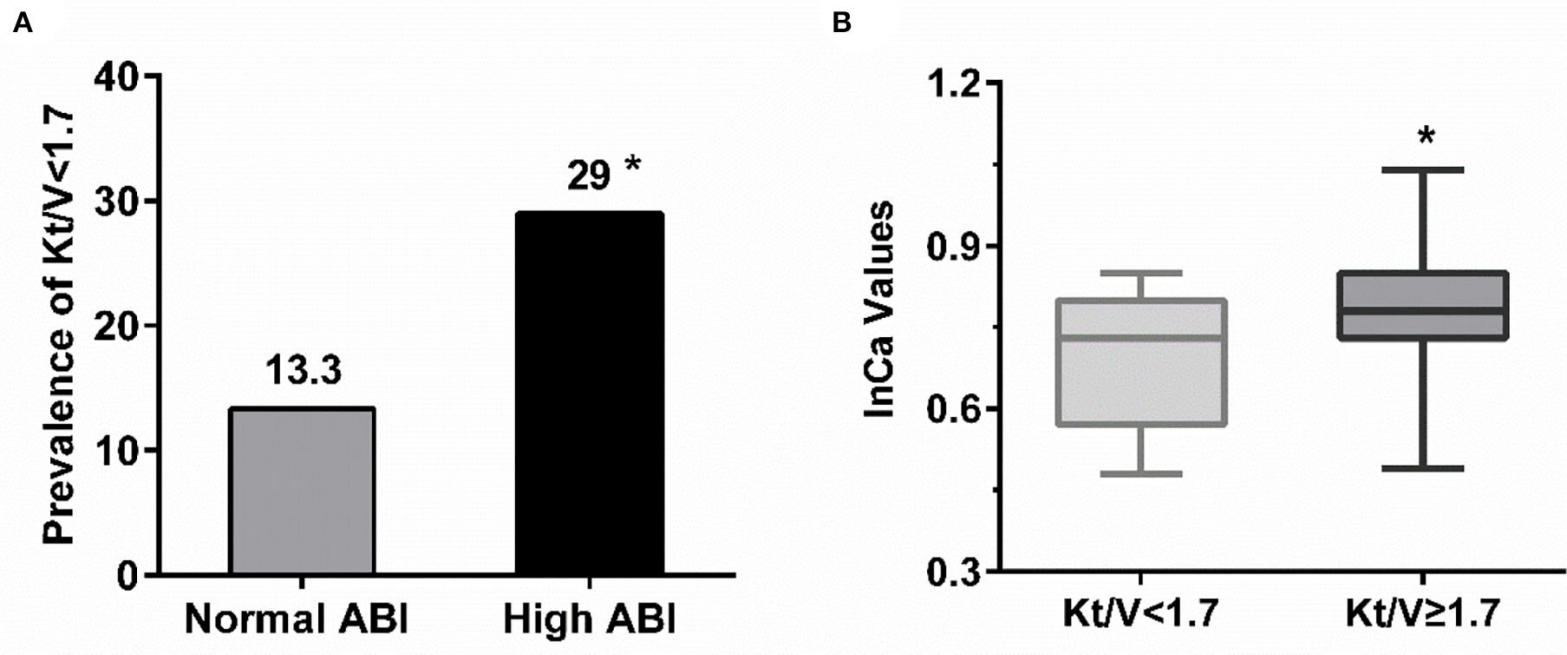
The relationship among adequacy dialysis and high ABI. **(A)** PD patient with high ABI tended to have Kt/V lower than 1.7 compared with those with normal value. *P < 0.05 vs. normal ABI group. **(B)** The level of serum calcium in PD patients with Kt/V ≥ 1.7 were relatively higher but within normal range. *P < 0.05 vs. Kt/V <1.7. ABI, ankle-brachial index; Ca, calcium.

**Figure 5:**
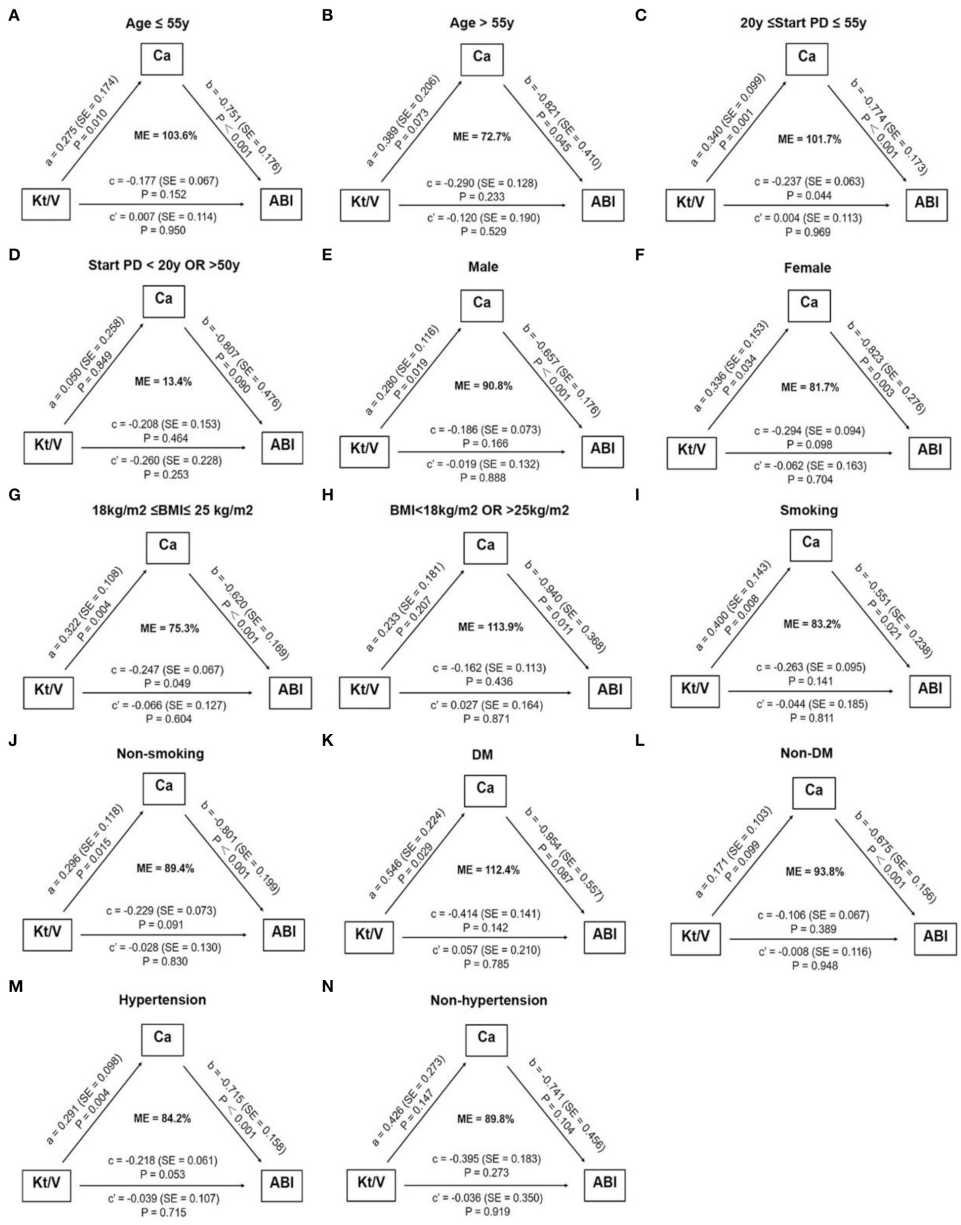
Subgroup analysis of assessing the mediation effect of serum calcium. The mediation effect of serum calcium on the association between Kt/V and high ABI only existed in PD patients starting dialysis before 55 years of age **(C)** and with normal BMI **(G)**. No mediation effect of serum calcium was observed in other subgroups **(A,B,D–F,H–N)**. The number of patients in subgroups: age ≤ 55 y: *n* = 88, Age > 55 y: *n* = 26; 20 y ≤ start PD ≤ 55 y: *n* = 93, start PD > 20 y OR <50 y: *n* = 17; male: *n* = 73, female: *n* = 41; 18 kg/m^2^ ≤ BMI ≤ 25 kg/m^2^: *n* = 79, BMI <18 kg/m^2^ OR > 25 kg/m^2^: *n* = 31; smoking: *n* = 45, non-smoking: *n* = 69; DM: *n* = 16, non-DM: *n* = 98; hypertension: *n* = 100, non-hypertension: *n* = 14. ABI, ankle-brachial index; BMI, body mass index; Ca, calcium; DM, diabetes mellitus; e-GFR, estimated-glomerular filtration rate; ME, mediation effect; PD, peritoneal dialysis; SE, standard error.

For PD patients, e-GFR was no longer suitable for evaluating kidney function. Instead, Kt/V was usually used to estimate dialytic adequacy. Kt/V ≥ 1.7 indicated dialytic adequacy while Kt/V <1.7 suggested inadequacy. About 29% of PD patients with high ABI had Kt/V lower than 1.7, which was significantly higher than those with normal ABI (29.0 vs. 13.3%, *P* = 0.031) (Figure 4A). Moreover, PD patients with Kt/V <1.7 tended to have lower Ca level compared to those with Kt/V ≥ 1.7 (0.69 ± 0.122 vs. 0.78 ± 0.11, *P* = 0.001) (Figure 4B). As Ca was the risk factor for high ABI in PD patients, we explored whether Ca mediated the association between Kt/V and ABI in PD patients. In PD patients, especially over 30 years old, the indirect effect of Ca with Kt/V became significant (ab = −0.215, 95% CI: −0.371 to −0.059, *P* = 0.006). Moreover, the total effect of Kt/V on ABI became non-significant after including Ca in the model (total effect c = −0.224, *P* = 0.036; direct effect c' = −0.034, *P* = 0.734). This indicated that ~86.2% of the relationship between Kt/V and ABI was mediated by Ca. The finding showed that Ca acted as a mediator in the relationship between Kt/V and ABI in PD patients (Figure 3B). Further, we conducted a subgroup analysis to identify the PD subgroups with high ABI mediated by Ca. As shown in Figure 5, the mediated effect of Ca became significant in PD patients starting PD before 55 years of age (ME = 101.7%, ab = −0.263, 95% CI: −0.452 to −0.074, *P* = 0.006) (Figure 5C) and with normal BMI (ME = 75.3%, ab = −0.200, 95% CI: −0.369 to −0.031, *P* = 0.020) (Figure 5G). However, the mediating effect did not exist in other subgroups (all *P* > 0.05).

## Discussion

Our study mainly found that the prevalence of high ABI, a strong MACE indictor, was increased with CKD progression. Interestingly, the prevalence of high ABI in PD patients was not further increased compared with non-dialyzed ones, which was possibly related to PD adequacy. Further mediation analysis revealed the important mediated effect of Ca on the relationship between PD adequacy and high ABI, especially in patients starting dialysis before 55 years of age and with normal BMI.

Although the development of effective dialysis and medication treatment for dialysis patients have been improved, CVD mortality is still predicted to be further increased by 2030, which causes great concern worldwide (1). In the late stages of CKD, many medications lack efficacy. For example, statins are supposed to be beneficial for the reduction of CVD mortality in non-dialyzed patients while similar protective effects cannot be seen in dialysis patients (26, 27). It indicates that other nontraditional risk factors may also contribute to the development of CVD in the dialysis population. As an important nontraditional risk factor, VC is supposed to be associated with incremental CVD mortality (6, 7). Many studies have demonstrated that the prevalence of VC is higher in CKD patients and elevates with CKD progression (28, 29). Likewise, our results also reveal that the prevalence of high ABI, predicting VC, increases with the decline in e-GFR levels. But intriguingly, the prevalence of high ABI is not further increased among PD patients. It seems that major risk factors influencing VC in PD patients are different from non-dialyzed ones. As PD patients with high ABI tend to have increased risk of MACE, the possible contributors of VC in PD patients warrant further exploration.

Numerous factors have been reported to contribute to VC in CKD patients, among which abnormal mineral metabolism is the most important contributor (30). As known, the kidney is a major organ regulating mineral balance. Thus, impaired kidney function frequently develops a deficiency of mineral metabolism, and the condition becomes the worst at the end-stage of CKD. Mineral disorder is commonly seen in dialysis patients, who are also more prevalent with VC (31). Importantly, abnormal mineral metabolism has been demonstrated to contribute to the large burden of CVD in dialysis patients (32). The additional health risks imposed by abnormal mineral metabolism are greatly challenging the management of patients with CKD although the traditional risk factors have well been controlled, especially for dialysis patients (8, 31). In this study, we observe that serum Ca, rather than traditional risk factors, plays a critical role in high ABI among PD patients. It seems that Ca balance might be a major contributor to VC after patients started PD (33). In fact, the essential role of Ca in the development of VC has been well elucidated (34). Growing evidence from randomized controlled trials demonstrates that more progression of VC and higher mortality are observed in CKD patients receiving calcium-based phosphate binders than those receiving non-calcium-containing phosphate binders because of the high risk of Ca overload (35, 36). Therefore, well-controlled Ca balance is critical for CKD patients, especially for dialysis patients with markedly reduced urinary Ca excretion.

Besides using drugs such as phosphate binders and vitamin D, adequate dialysis is a specific and effective way to maintain mineral balance for dialysis patients. As known, inadequate dialysis contributes greatly to increased risk of all-cause and CVD mortality in dialysis patients (37). Dialysis adequacy also plays a role in the development of VC. Estimated by low Kt/V level, inadequate dialysis is reported to be an independent risk factor for VC in dialysis patients (38, 39). Likewise, we find that the Kt/V level of PD patients with high ABI tends to be <1.7 and PD adequacy is associated with a lower prevalence of high ABI. This might be because insufficient PD could lead to internal environmental disorders and mineral metabolism imbalance, which then initiates vascular lesions and VC (5, 31). Indeed, further mediation analysis estimates that ~84.4% of the relationship between Kt/V and ABI might be mediated by Ca in PD patients. It suggests that maintaining neutral Ca balance after adequate dialysis is important for reducing the prevalence of high ABI for PD patients. It might be another reason to explain why recent studies have reported that PD patients seem to face additional CVD risk (14). Therefore, dialysis treatment for PD patients should be individualized after comprehensive assessments, which include both the adequacy of dialysis and the level of post-dialysis Ca. Nevertheless, due to the limited data, further investigation is needed to verify our results.

Another interesting result we discovered is that the mediation effect of Ca only exists in PD patients starting dialysis before 55 years of age and with normal BMI. It seems that only in such relatively low-risk PD patients, adequate dialysis could retard the development of VC by maintaining a neutral Ca balance. This might explain why some PD patients with adequate dialysis also suffer from high ABI, as both age and abnormal nutritional status are important contributors to VC in CKD patients (30, 40). With a series of traditional cardiovascular risks, PD patients tend to suffer from advanced VC, and even adequate dialysis could not bring further benefits to its regression. Therefore, initiating PD earlier than 55 years of age and keeping BMI in the normal range may help patients benefit more from dialysis adequacy.

There are several limitations in this study. First, this is a cross-sectional, observational study. It might be impossible to fully confirm the relationship among dialysis adequacy, Ca, and high ABI. But we conduct the medication analysis which is used to analyze the statistically causal relationship. Second, the number of PD patients enrolled is relatively small and selection bias exists as patients without the contraindications of PD are included in this study. Therefore, further prospective studies with a larger sample size are needed to verify our findings. Third, we used ABI to estimate VC instead of computed tomography (CT) scanning. Although CT is the most common method used to assess VC, it may be not suitable for CKD patients because of the renal damage of the contrast agent. As a simple and noninvasive method, ABI has no renal damage and is more convenient and economical for CKD patients in systematic VC detection.

In conclusion, we provide clinical evidence that PD inadequacy correlated with increased high ABI occurrence, which predicts incremental risk of MACE, and that Ca might be an important mediator, especially for those starting PD before 55 years of age and with normal BMI. Improvement of PD adequacy by controlling neutral Ca balance seems to be promising to reduce the risk of MACE evaluated by high ABI for patients on PD.

## Data Availability Statement

The raw data supporting the conclusions of this article will be made available by the authors, without undue reservation.

## Ethics Statement

The studies involving human participants were reviewed and approved by the Ethics Committee of the Tungwah Hospital of Sun Yat-sen University. The patients/participants provided their written informed consent to participate in this study.

## Author Contributions

HH contributed to conception and design of the study and the analyses. XS, WH, MZ, and YZ participated in data collection. WH contributed to the statistical analysis and the draft manuscript. LZ, JC, and HH reviewed the manuscript and made final changes. All authors contributed to the article and approved the submitted version.

## Funding

This work was supported in part by National Key Research and Development Program (2020YFC2004405), National Natural Science Foundation of China (NSFC) (82073408) to JC, NSFC (82061160372 and 81870506), project of traditional Chinese medicine in Guangdong province (20201062), Basic Research Project of Shenzhen Science and Technology Innovation Committee (JCYJ20180306174648342 and JCYJ20190808102005602), Futian District Public Health Scientific Research Project of Shenzhen (FTWS2019003) and Shenzhen Key Medical Discipline Construction Fund (SZXK002), and Guangdong Basic and Applied Basic Research Foundation (2021B1515120083) to HH; Natural Science Foundation of Guangdong Province (A2016248) and Guangdong Basic and Applied Basic Research Foundation (2020B1515120037) to XS.

## Conflict of Interest

The authors declare that the research was conducted in the absence of any commercial or financial relationships that could be construed as a potential conflict of interest.

## Publisher's Note

All claims expressed in this article are solely those of the authors and do not necessarily represent those of their affiliated organizations, or those of the publisher, the editors and the reviewers. Any product that may be evaluated in this article, or claim that may be made by its manufacturer, is not guaranteed or endorsed by the publisher.

## References

[1] Webster AC Nagler EV Morton RL Masson P. Chronic kidney disease. Lancet. (2017) 389:1238–52. 10.1016/S0140-6736(16)32064-5 27887750

[2] Benjamin EJ Virani SS Callaway CW Chamberlain AM Chang AR Cheng S. Heart disease and stroke statistics-2018 update: a report from the american heart association. Circulation. (2018) 137:e67–e492. 10.1161/CIR.0000000000000558 29386200

[3] Ortiz A Covic A Fliser D Fouque D Goldsmith D Kanbay M. Epidemiology, contributors to, and clinical trials of mortality risk in chronic kidney failure. Lancet. (2014) 383:1831–43. 10.1016/S0140-6736(14)60384-6 24856028

[4] Bozic M Mendez-Barbero N Gutierrez-Munoz C Betriu A Egido J Fernandez E. Combination of biomarkers of vascular calcification and sTWEAK to predict cardiovascular events in chronic kidney disease. Atherosclerosis. (2018) 270:13–20. 10.1016/j.atherosclerosis.2018.01.011 29407881

[5] Scialla JJ Kao WH Crainiceanu C Sozio SM Oberai PC Shafi T. Biomarkers of vascular calcification and mortality in patients with ESRD. Clin J Am Soc Nephrol. (2014) 9:745–55 10.2215/CJN.05450513 PMC3974354 24458076

[6] Chen J Budoff MJ Reilly MP Yang W Rosas SE Rahman M. Coronary artery calcification and risk of cardiovascular disease and death among patients with chronic kidney disease. JAMA Cardiol. (2017) 2:635–43. 10.1001/jamacardio.2017.0363 28329057 PMC5798875

[7] Grams ME Yang W Rebholz CM Wang X Porter AC Inker LA. Risks of adverse events in advanced CKD: the chronic renalinsufficiency cohort (CRIC) study. Am J Kidney Dis. (2017) 70:337–46. 10.1053/j.ajkd.2017.01.050 28366517 PMC5572665

[8] Mizobuchi M Towler D Slatopolsky E. Vascular calcification: the killer of patients with chronic kidney disease. J Am Soc Nephrol. (2009) 20:1453–64. 10.1681/ASN.2008070692 19478096

[9] Fowkes FG Murray GD Butcher I Heald CL Lee RJ Chambless LE. Ankle brachial index combined with Framingham Risk Score to predict cardiovascular events and mortality: a meta-analysis. JAMA. (2008) 300:197–208. 10.1001/jama.300.2.197 18612117 PMC2932628

[10] Hendriks EJ Westerink J de Jong PA de Borst GJ Nathoe HM Mali WP. Association of high ankle brachial index with incident cardiovascular disease and mortality in a high-risk population. Arterioscler Thromb Vasc Biol. (2016) 36:412–7. 10.1161/ATVBAHA.115.306657 26715681

[11] Adragao T Pires A Branco P Castro R Oliveira A Nogueira C. Ankle–brachial index, vascular calcifications and mortality in dialysis patients. Nephrol Dial Transplant. (2012) 27:318–25. 10.1093/ndt/gfr233 21551082

[12] Arroyo D Betriu A Valls J Gorriz JL Pallares V Abajo M. Factors influencing pathological ankle-brachial index values along the chronic kidney disease spectrum: the NEFRONA study. Nephrol Dial Transplant. (2017) 32:513–20. 10.1093/ndt/gfw039 27190385

[13] Tong J Liu M Li H Luo Z Zhong X Huang J. Mortality and associated risk factors in dialysis patients with cardiovascular disease. Kidney Blood Press Res. (2016) 41:479–87. 10.1159/000443449 27434642

[14] Bartosova M Schaefer B Bermejo JL Tarantino S Lasitschka F Macher-Goeppinger S. Complement activation in peritoneal dialysis-induced arteriolopathy. J Am Soc Nephrol. (2018) 29:268–82. 10.1681/ASN.2017040436 29046343 PMC5748916

[15] Maghbooli Z Shabani P Gorgani-Firuzjaee S Hossein-Nezhad A. The association between bone turnover markers and microvascular complications of type 2 diabetes. J Diabetes Metab Disord. (2016) 15:51. 10.1186/s40200-016-0274-2 27826545 PMC5100233

[16] Chan CT Blankestijn PJ Dember LM Gallieni M Harris DCH Lok CE. Dialysis initiation, modality choice, access, and prescription: conclusions from a Kidney Disease: Improving Global Outcomes (KDIGO) Controversies Conference. Kidney Int. (2019) 96:37–47. 10.1016/j.kint.2019.01.017 30987837

[17] KDOQI clinical practice guideline for hemodialysis adequacy: 2015 update. Am J Kidney Dis. (2015) 66:884–930. 10.1053/j.ajkd.2015.07.015 26498416

[18] II. NKF-K/DOQI clinical practice guidelines for peritoneal dialysis adequacy: update 2000. Am J Kidney Dis. (2001) 37:S65–136. 10.1016/S0272-6386(01)70006-6 11229968

[19] Blake PG Bargman JM Brimble KS Davison SN Hirsch D McCormick BB. Clinical practice guidelines and recommendations on peritoneal dialysis adequacy 2011. Perit Dial Int. (2011) 31:218–39. 10.3747/pdi.2011.00026 21427259

[20] Zhang Y Chen J Zhang K Kong M Wang T Chen R. Inflammation and oxidative stress are associated with the prevalence of high aankle-brachial index in metabolic syndrome patients without chronic renal failure. Int J Med Sci. (2013) 10:183–90. 10.7150/ijms.5308 23329891 PMC3547217

[21] Zhang Y Chen J Zhang K Wang T Kong M Chen R. Combination of high ankle-brachial index and hard coronary heart disease Framingham Risk Score in predicting the risk of ischemic stroke in general population. PLoS ONE. (2014) 9:e106251. 10.1371/journal.pone.0106251 25198106 PMC4157777

[22] Fihn SD Blankenship JC Alexander KP Bittl JA Byrne JG Fletcher BJ. 2014 ACC/AHA/AATS/PCNA/SCAI/STS focused update of the guideline for the diagnosis and management of patients with stable ischemic heart disease: a report of the American College of Cardiology/American Heart Association Task Force on Practice Guidelines, and the American Association for Thoracic Surgery, Preventive Cardiovascular Nurses Association, Society for Cardiovascular Angiography and Interventions, and Society of Thoracic Surgeons. Circulation. (2014) 130:1749–67. 10.1161/CIR.0000000000000095 25070666

[23] Ponikowski P Voors AA Anker SD Bueno H Cleland JG Coats AJ. 2016 ESC Guidelines for the diagnosis treatment of acute chronic heart failure: The Task Force for the diagnosis treatment of acute chronic heart failure of the European Society of Cardiology (ESC). Developed with the special contribution of the Heart Failure Association (HFA) of the ESC. Eur J Heart Fail. (2016) 18:891–975. 10.1002/ejhf.592 27207191

[24] Roffi M Patrono C Collet JP Mueller C Valgimigli M Andreotti F. 2015 ESC guidelines for the management of acute coronary syndromes in patients presenting without persistent ST-segment elevation: Task Force for the Management of Acute Coronary Syndromes in Patients Presenting without Persistent ST-Segment Elevation of the European Society of Cardiology (ESC). Eur Heart J. (2016) 37:267–315. 10.1093/eurheartj/ehv320 26320110

[25] MacKinnon DP Fairchild AJ Fritz MS. Mediation analysis. Annu Rev Psychol. (2007) 58:593–614. 10.1146/annurev.psych.58.110405.085542 16968208 PMC2819368

[26] Palmer SC Navaneethan SD Craig JC Johnson DW Perkovic V Hegbrant J. HMG CoA reductase inhibitors (statins) for people with chronic kidney disease not requiring dialysis. Cochrane Database Syst Rev. (2014) Cd007784. 10.1002/14651858.CD007784.pub2 24880031

[27] Palmer SC Navaneethan SD Craig JC Johnson DW Perkovic V Nigwekar SU. HMG CoA reductase inhibitors (statins) for dialysis patients. Cochrane Database Syst Rev. (2013) Cd004289. 10.1002/14651858.CD004289.pub5 PMC10754478 24022428

[28] Ichii M Ishimura E Shima H Ohno Y Ochi A Nakatani S. Quantitative analysis of abdominal aortic calcification in CKD patients without dialysis therapy by use of the Agatston score. Kidney Blood Press Res. (2013) 38:196–204. 10.1159/000355768 24732137

[29] Budoff MJ Rader DJ Reilly MP Mohler ER 3rd Lash J. Relationship of estimated GFR and coronary artery calcification in the CRIC (Chronic Renal Insufficiency Cohort) Study. Am J Kidney Dis. (2011) 58:519–26. 10.1053/j.ajkd.2011.04.024 21783289 PMC3183168

[30] Zhang K Gao J Chen J Liu X Cai Q Liu P. MICS, an easily ignored contributor to arterial calcification in CKD patients. Am J Physiol Renal Physiol. (2016) 311:F663–70. 10.1152/ajprenal.00189.2016 27335374

[31] Covic A Vervloet M Massy ZA Torres PU Goldsmith D Brandenburg V. Bone and mineral disorders in chronic kidney disease: implications for cardiovascular health and ageing in the general population. Lancet Diabetes Endocrinol. (2018) 6:319–31. 10.1016/S2213-8587(17)30310-8 29050900

[32] Lee SA Lee MJ Ryu GW Jhee JH Kim HW Park S. Low serum intact parathyroid hormone level is an independent risk factor for overall mortality and major adverse cardiac and cerebrovascular events in incident dialysis patients. Osteoporos Int. (2016) 27:2717–26. 10.1007/s00198-016-3636-1 27216997

[33] Abe M Okada K Soma M. Mineral metabolic abnormalities and mortality in dialysis patients. Nutrients. (2013) 5:1002–1023. 10.3390/nu5031002 23525083 PMC3705332

[34] Moe SM. Calcium as a cardiovascular toxin in CKD-MBD. Bone. (2017) 100:94–9. 10.1016/j.bone.2016.08.022 27576942 PMC5329167

[35] Patel L Bernard LM Elder GJ. Sevelamer versus calcium-based binders for treatment of hyperphosphatemia in CKD: a meta-analysis of randomized controlled trials. Clin J Am Soc Nephrol. (2016) 11:232–44. 10.2215/CJN.06800615 26668024 PMC4741042

[36] Elder GJ Center J. The role of calcium and non calcium-based phosphate binders in chronic kidney disease. Nephrology. (2017) 22 (Suppl. 2):42–6. 10.1111/nep.13031 28429551

[37] Parker TF 3rd Husni L Huang W Lew N Lowrie EG. Survival of hemodialysis patients in the United States is improved with a greater quantity of dialysis. Am J Kidney Dis. (1994) 23:670–80. 10.1016/S0272-6386(12)70277-9 8172209

[38] Schlieper G Brandenburg V Djuric Z Damjanovic T Markovic N Schurgers L. Risk factors for cardiovascular calcifications in non-diabetic Caucasian haemodialysis patients. Kidney Blood Press Res. (2009) 32:161–8. 10.1159/000221064 19468238

[39] Wu CF Lee YF Lee WJ Su CT Lee LJ Wu KD. Severe aortic arch calcification predicts mortality in patients undergoing peritoneal dialysis. J Formos Med Assoc. (2017) 116:366–72. 10.1016/j.jfma.2016.06.006 27497908

[40] Okamoto T Hatakeyama S Kodama H Horiguchi H Kubota Y Kido K. The relationship between maintaining neutral calcium balance and progression of aortic calcification in patients on maintenance hemodialysis. BMC Nephrol. (2018) 19:71. 10.1186/s12882-018-0872-y 29558928 PMC5861641

