# Supplementary material for: Abnormal Calcium Metabolism Mediated Increased Risk of Cardiovascular Events Estimated by High Ankle-Brachial Index in Patients on Peritoneal Dialysis
Source: Front Cardiovasc Med. 2022 Jul 28;9:920431. doi: 10.3389/fcvm.2022.920431 (PMC9369253; doi:10.3389/fcvm.2022.920431)
Supplement: Supplementary file 1 [file Table_1.docx]

Supplementary Material

# Supplementary Tables

**Supplementary Table 1**. Comparison of the demographic and biochemical data between high ABI and normal ABI groups.

|  | Normal ABI group | High ABI group | *P* |
| --- | --- | --- | --- |
| Number(n)  Age(year)  Male gender (%)  BMI (kg/m^2^)  SBP(mm/Hg)  DBP(mm/Hg)  Hypertension(%)  Smoking(%)  DM(%)  UA (μmol/L)  FPG(mmol/L)  ALP(U/L)  Ca (mmol/L)  P (mmol/L)  K(mmol/L)  TC(mmol/L)  LDL-C(mmol/L)  HDL-C(mmol/L)  TG(mmol/L)  Cr (μmol/L)  CysC(mg/L)  RRF [ml/(min·1.73m^2^)]  PD period(month) | 83  42±13  61.4  21.95±3.86  150.48±20.99  89.84±14.01  86.7  41  9.6  436.76±93.82  4.96(4.37-5.30)  73 (60-81)  2.24±0.20  1.64±0.56  4.05±0.78  4.84±1.35  2.63(2.10-3.08)  1.25±0.40  1.26(0.83-2.45)  1000.40±331.59  5.86±1.63  7.34±3.33  39.30±28.15 | 31  51±16  71  22.75±3.40  157.26±21.57  84.94±11.18  90.3  35.5  25.8  415.97±106.90  5.14(4.73-6.03)  79 (68-94)  1.93±0.18  1.95±0.44  4.03±0.67  4.57±0.91  2.71(2.23-2.92)  1.23±0.36  1.21(1.03-1.86)  924.42±293.83  5.62±1.26  7.06±2.77  37.52±26.11 | 0.007*  0.346  0.309  0.131  0.083  0.605  0.594  0.027*  0.313  0.041*  0.007*  0.001*  0.002*  0.889  0.303  0.977  0.769  0.921  0.265  0.460  0.352  0.345 |

ABI, ankle-brachial index; ALP, alkaline phosphatase; BMI, body mass index; Ca, calcium; CKD, chronic kidney disease; Cr, creatinine; CysC, cystatin C; DBP, diastolic blood pressure; DM, diabetes mellitus; e-GFR, estimated-glomerular filtration rate; FPG, fasting plasma glucose; HDL-C, high-density lipoprotein cholesterol; K, serum potassium; LDL-C, low-density lipoprotein cholesterol; P, phosphorus; PD, peritoneal dialysis; SBP, systolic blood pressure; TC, total cholesterol; TG, triglycerides. **P* < 0.05.
